# Validity of two physical activity questionnaires (IPAQ and PAQA) for Vietnamese adolescents in rural and urban areas

**DOI:** 10.1186/1479-5868-5-37

**Published:** 2008-07-10

**Authors:** Carl K Lachat, Roosmarijn Verstraeten, Le Nguyen Bao Khanh, Maria Hagströmer, Nguyen Cong Khan, Nguyen Do Anh Van, Nguyen Quang Dung, Patrick W Kolsteren

**Affiliations:** 1 Nutrition and Child Health Unit, Prince Leopold Institute of Tropical Medicine, Antwerp, Belgium; 2 Department of Food Safety and Food Quality, Faculty of Bioscience Engineering, Ghent University, Belgium; 3 National Institute of Nutrition, Hanoi, Vietnam; 4 Unit for Preventive Nutrition, Department of Biosciences and Nutrition, Karolinska Institutet, Stockholm, Sweden

## Abstract

**Background:**

Although physical activity is recognised to be an important determinant of health and nutritional status, few instruments have been developed to assess physical activity in developing countries. The aim of this study was to compare the validity of the short form of the International Physical Activity Questionnaire (IPAQ) and a locally adapted version of the Physical Activity Questionnaire for Adolescents (PAQA) for use in school going adolescents in rural and urban areas in Vietnam.

**Methods:**

Sixteen year old adolescents from rural areas (n = 137) and urban areas (n = 90) completed the questionnaires in 2006. Test-retest reliability was assessed by comparing registrations after 2 weeks. Criterion validity was assessed by comparison with 7 days continuous accelerometer logging. Validity of the two methods was assessed using Spearman correlation coefficient, intra class correlation coefficients (ICC) and Kappa statistics.

**Results:**

Reliability of both questionnaires was poor for both the IPAQ (ICC = 0.37) and the PAQA (ICC = 0.40). Criterion validity of both questionnaires was acceptable and similar for the IPAQ (ρ = 0.21) and the PAQA (ρ = 0.27) but a significantly lower validity was observed in rural areas. Both forms poorly estimated time spent on light, moderate and vigorous physical activity. Agreement of both questionnaires to classify individuals was also low but the IPAQ performed better than the PAQA.

**Conclusion:**

Both questionnaires have a similar and overall poor validity to be used as a population instrument in Vietnam. Low reliability and classification properties in rural areas call for further research for specific use in such settings.

## Background

Parallel to demographic changes, nutrition and lifestyle patterns are undergoing serious changes in many countries of the world. Diets are gradually becoming richer in saturated fat and refined foods, while fruit and vegetable consumption and physical activity levels are believed to be decreasing [1]. The nutritional status of adolescents is known to be a powerful predictor for diet-related chronic diseases independent of adult weight [2,3]. Healthy or unhealthy lifestyles may develop in adolescence and persist further on in life [4,5]. Lifestyle modifications, in particular a healthy diet and an appropriate level of physical activity, remain the foundation for the prevention of diet related non-communicable diseases [4,6].

In Asia, economic development and urbanisation is taking place rapidly [5,7-10]. Vietnam has experienced fast economic growth and urbanisation after the economic reforms of the mid 1980's. Childhood malnutrition has been the major concern for public health nutrition for decades but a number of studies show the emerging new burden of non communicable nutrition related diseases [11-13].

Although the promotion of physical activity is at the centre stage of health promotion activities [5,14], few studies have assessed instruments to measure physical activity of adolescents in developing countries. Questionnaires are commonly accepted as instruments for population-based studies for this [15,16]. To evaluate their criterion validity, questionnaire registrations are usually compared to energy expenditure as assessed by double labelled water or registrations by motion sensors such as accelerometers. Compared to the use of double labelled water, the use of motion sensors has the advantage of being a cheap, simple and non-invasive technique appropriate for use in larger groups of people. Accelerometer based assessment of physical activity nevertheless presents considerable challenges with regard to financial costs, technical know-how and supervision, which makes that questionnaires are still predominantly used in developing countries as epidemiological instrument.

The International Physical Activity Questionnaire (IPAQ) has been validated for use in adult population studies in various countries, but its validity for use in developing countries and adolescents needs further research [17,18]. The IPAQ is largely a self-reported questionnaire that records duration of different levels of physical activity for a habitual or past week. The IPAQ has a long and a short version which have a similar validity [17]. The short version is a dimension-based instrument. The questionnaire is structured to capture physical activity in 4 generic dimensions of physical activity, namely vigorous, moderate, walking and sitting.

The IPAQ has been modified to measure physical activity during school periods into a Physical Activity Questionnaire for Adolescents (PAQA). The PAQA is an activity-based questionnaire and measures the frequency and the duration of activities during specific moments of the day. Comparison of the PAQA against double labelled water measurements however, showed that it was unable to predict energy expenditure well, mainly due to the large amount of time not captured by the questionnaire [19].

Changes in lifestyles in adolescents living in developing countries call for sound monitoring and surveillance instruments [14]. Ideally, such instruments need to be valid in both rural and urban areas. Since lifestyles and physical activity patterns in rural and urban areas are potentially different, it is unclear to what extent existing instruments can be applied in both settings.

## Objective

The overall objective of this study is to test two existing instruments for the assessment of physical activity in adolescents in Vietnam. More specifically, we aimed to test the reliability and criterion validity of the short version of the IPAQ and a locally modified PAQA questionnaire for use in school going adolescents and to compare their validity in rural and urban areas. To our knowledge, no other studies have aimed to validate physical activity questionnaires in adolescents in developing countries with accelerometers.

## Methods

Both questionnaires were tested for reliability through test-retest comparison. Criterion validity of these questionnaires was evaluated using accelerometers, which have been validated previously as instruments to measure physical activity in adolescents and children in developed countries [20-24] and suggested as an appropriate criterion measure for the validation of physical activity questionnaires [25]. As a third measure of validity, groups of different levels of physical activity were defined and the classification properties of the instruments were compared.

Vietnamese adolescents may be shorter than other participants in international studies, which may have lead to different acceleration patterns. No studies have currently looked into the effect of stature on the validity of accelerometer data and its cut-off values as objective measure of physical activity. We also analysed the effect of Body Mass Index (BMI) on the validity of the instruments.

### Sampling

Data was collected from a convenience sample of 275 adolescents of 16 years of age (Grade 11) from 3 classes in a school in an urban area of Hanoi and 5 classes of a rural school in Ha Nam province, North Vietnam. Schools were selected on the basis of willingness to cooperate in the study and absence of health or nutrition related interventions which could bias the results. There were no exclusion criteria. Due to the limited number of accelerometers available, a sub-sample of the study population, 193 children (70%), were randomly selected to wear the instruments.

### Data collection

The MTI Actigraph GT256 accelerometers (Manufacturing Technology Incorporated, Fort Walton Beach FL) were used and were programmed to register 1-minute cycles. Adolescents were asked to wear the accelerometer for 8 consecutive days during waking hours and to remove it during showering, bathing or swimming. The accelerometer was attached on a belt and worn on the right side of the hip in accordance to the guidelines suggested by Trost *et al *[26,27]. Each participant received a personal demonstration by trained professionals and written instructions on how to wear the accelerometer. In addition, a registration card was distributed to remind the students to remove the accelerometer before bathing and putting it back on afterwards.

The IPAQ and the PAQA were self-reported and referred to a habitual week. They were completed twice with a two-week interval. The questionnaires were randomly presented to the participants to avoid systematic bias of filling the same questionnaire first.

Prior to the study, the short form of the IPAQ was translated from English to Vietnamese, checked through back translations and pre-tested in a classroom. The questionnaires were adapted until the form was clear to all participants. Specific questions to capture time spent sitting and sleeping were added. Published instructions for analysis and data management were followed to compute the IPAQ variables [28]. The IPAQ outcome variables used in the study are the estimate of total physical activity as metabolic equivalent (MET) expressed as MET-minutes.day^-1 ^(IPAQ_TOT_), the average duration of sitting and sleeping (IPAQ_LPA_), moderate (IPAQ_MPA_) and vigorous (IPAQ_VPA_) activities per day.

The PAQA was adapted to the Vietnamese context and further modified to reduce underreported time. To do so, the key moments of time expenditure in a habitual week were identified by the adolescents during focus group discussions. These moments were physical activity classes at school, class breaks, transportation to and from school, recreation times, attending lectures and sleeping. Specific questions were incorporated in the PAQA form to register time and activities during these moments. Given the importance of extra classes outside official school hours, specific questions were included to capture physical activities at that time. The different elements of the PAQA questionnaire are shown in Table 1. Time recordings were subsequently multiplied by specific MET values for each activity [29]. A total measure of physical activity (PAQA_TOT_) was obtained by summing all MET values for each dimension of physical activity. By grouping different activities according to their intensity level, the average duration per day for sitting and sleeping (PAQA_LPA_), moderate (PAQA_MPA_) and vigorous (PAQA_VPA_) physical activity was obtained.

**Table 1:** Elements of the PAQA

**Part 1: Physical activity in official school and extra school classes**
▪Frequency and duration of activities during physical education classes during a habitual week with assessment of the intensity: sweating and raise of heart frequency highly above normal, intensities that induce slightly sweating and raise heart frequency slightly above normal, intensities that do not induce sweating or raise heart frequencies or rarely participate
▪Number of days of extra-classes in a habitual week
▪Duration of the gaps between lessons during one whole habitual school week, during official classes and extra school classes with assessment of intensity of activities during the gaps between classes (sitting or standing, walking or specific sports)
▪Duration of sitting in classes on a habitual weekday during official classes, extra school classes and on a weekend-day

**Part 2: Physical activity during transportation to and from school and the extra school classes**

▪Frequency and duration of walking briskly for more than 10 minutes to get to and from the school and extra school classes
▪Frequency and duration of biking briskly for more than 10 minutes to get to and from the school and extra school classes
▪Frequency and duration of motorised transport to get to and from the school and extra school classes

**Part 3: Physical activity during spare time**

▪Frequency, duration and name of activities that last at least 10 minutes at high intensity
▪Frequency, duration and name of activities that last more than 10 minutes at moderate intensity
▪Frequency and duration of walking briskly more than 10 minutes for pleasure or exercise
▪Frequency and duration of motorised transport to get from one place to another
▪Frequency and duration of sitting during a week-day and weekend day
▪Type, frequency and duration of leisure time activities and low physical activity not mentioned earlier

**Part 4: Sleeping**

▪Duration of sleeping at night and day during a habitual week-day
▪Duration of sleeping at night and day during weekend-day

Weight was recorded wearing light clothing and no shoes using a digital scale (Seca Uniscale Germany) up to 100 g. Height was measured using a portable fixed base stadiometer (CMS Weighing Equipment, UK) to 0.1 cm. Weight and height were measured by a trained nurse and recorded in double by the researchers. Categories of BMI were computed using the references for adolescents as proposed by Cole *et al *[30]. The methods and objective of the study were explained to adolescents and written consent from their parents was obtained. The study protocol was approved by the Medical Research Ethics Committee of the National Institute of Nutrition of Vietnam.

### Data reduction and analysis

Accelerometers were considered malfunctioning if no counts were registered during an entire day or if a constant number of counts was registered per second for a whole day. Accelerometer data reduction was carried out in a program written in VisualBasic.NET using specifically developed software by the Karolinska Institute in Sweden.

Accelerometer data recorded on the first day (day of initiation) and last day (day of termination) were excluded from analysis. Outcome variables computed were: total counts per day (ACC_TOT_) and average time spent on light (ACC_LPA_), moderate (ACC_MPA_) and vigorous (ACC_VPA_) physical activities. Age-specific count ranges as proposed by Trost *et al *[31] and reviewed by Anderson *et al *[32] were used to discriminate activity levels as registered by the accelerometer. Estimations of physical activity by the questionnaires and the accelerometers were standardised to 24 hours to account for the total volume of physical activity reported.

The IPAQ and PAQA data were entered in Epidata and verified using double data entry. All analysis was carried out using Intercooled Stata v8.0 (Statacorp, College station, Texas, USA) with α = 0.05. As the physical activity data are commonly not normally distributed, results are presented here as median values. Continuous data were transformed to normality using Stata command lnskew0. All tests were two-sided.

Test-retest reliability was examined using both Spearman and intra class correlation coefficients. Criterion validity was assessed using Spearman correlation coefficients. The total counts of the accelerometer were correlated with the total MET's measured by the questionnaires. Based on the cut-offs for low, moderate and vigorous activities, the time spent on each of those was measured by the accelerometer and correlated to the respective times as measured by the questionnaires. Classification agreement of both test-retest and criterion validity was further examined using Kappa tests, based on groups defined by tertiles of IPAQ_TOT_, PAQA_TOT _and ACC_TOT_.

### Feasibility

Focus group discussions with the students were conducted to assess the experiences of the participants with filling both questionnaires and wearing the accelerometer. Each student also completed an evaluation form to collect feedback on wearing the accelerometer.

## Results

The IPAQ forms were completed by 227 students and 200 (88%) of them completed the form during the second survey. The PAQA data of 2 classes were excluded from analysis since these forms did not contain two questions that were added later to improve the PAQA. In total, 158 children provided valid data for the PAQA for the first survey and 200 students for the second one. After excluding data from malfunctioning accelerometers (n = 5), 188 students provided valid accelerometer data. Participants from urban areas had a higher weight (P < 0.001), height (P = 0.0016) and BMI (P < 0.001) compared to those from rural areas. Male students were taller (P < 0.0001), heavier (P < 0.0001) and had a higher BMI (P = 0.0084) compared to female students. On average, the participants were 16 years ± 0.4 old. The total time recorded by the accelerometers was on average 17 hours and 48 minutes per day. Seven children (3%) were overweight and none were obese. All the overweight children were male adolescents living in urban areas.

BMI, weight, IPAQ_TOT _and PAQA_TOT _of the sub-sample of children that wore the accelerometer was not different from those that did not wear the accelerometer (P = 0.58, P = 0.09, P = 0.55 and P = 0.55 respectively).

A descriptive summary of the main outcome variables is included in Table 2. The values tabulated are those obtained from the first survey of the IPAQ and the PAQA. Children in urban areas were found to be marginally more active compared to their peers in rural areas according to the questionnaires but not for the accelerometer readings.

**Table 2:** Descriptives of accelerometer, the IPAQ and the PAQA measurements (Median values and 95%CI)

	**All**			**Rural**			**Urban**			**P**^1^
				
	**Median**	**95% CI**		**Median**	**95% CI**		**Median**	**95% CI**		
							
**IPAQ**										
IPAQ_TOT_^4^	1107	1042	1181	1045	1015	1167	1166	1054	1350	0.03^2^
IPAQ_LPA_^5^	1351	1335	1365	1346	1330	1362	1356	1334	1377	0.62^2^
IPAQ_MPA_^5^	36	30	47	43	33	53	34	24	51	0.83^3^
IPAQ_VPA_^5^	9	5	16	4	2	9	18	8	30	0.01^3^

**PAQA**										

PAQA_TOT_^4^	2551	2477	2640	2522	2427	2615	2590	2463	2762	0.03^2^
PAQA_LPA_^5^	988	947	1021	988	949	1018	986	895	1074	0.72^2^
PAQA_MPA_^5^	92	84	107	95	84	121	89	75	114	0.82^2^
PAQA_VPA_^5^	15	13	16	14	12	15	16	13	18	0.03^3^

**Accelerometer**										

ACC_TOT_^6^	281272	269650	299521	292384	275432	334176	269665	238863	285869	0.11^3^
ACC_LPA_^5^	1411	1407	1416	1410	1401	1417	1412	1407	1418	0.80^2^
ACC_MPA_^5^	27	23	30	28	20	33	26	21	31	0.91^2^
ACC_VPA_^5^	1	0	1	1	0	1	1	0	2	0.44^3^

^1 ^Comparison between urban and rural areas;

^2 ^Standard t-test;

^3 ^Non-parametric Wilcoxon rank-sum test;

^4 ^MET-minutes.day^-1^; ^5^Minutes.day^-1^; ^6^Total counts.day^-1^

On average, both the IPAQ and the PAQA produced higher estimations for the duration of MPA and VPA compared to the accelerometer in both rural and urban areas. In total, 46% of the participants were moderately active for at least 30 minutes per day as measured by the accelerometer. These percentages are 78% of the participants as measured by the IPAQ and 94% by the PAQA. For the duration of moderate activity for at least 60 minutes, these percentages are 16%, 59% and 84% for the accelerometer, the IPAQ and the PAQA assessments respectively.

### Reliability

Table 3 shows how the overall reliability of total physical activity measurement was comparable for the IPAQ and the PAQA. The IPAQ demonstrated lower reliability and the PAQA higher reliability in urban areas. Analysis of the different dimensions of physical activity showed lower reliability for measurements of durations of LPA and MPA. Reliability of time spent on MPA was particularly poor in urban areas for IPAQ and rural areas for PAQA.

**Table 3:** Reliability of the IPAQ and PAQA questionnaires (Spearman coefficient ρ and intra class correlation coefficients ICC)

	**All**		**Rural**		**Urban**	
			
	**ρ**	**ICC**	**ρ**	**ICC**	**ρ**	**ICC**
			
**IPAQ**						
IPAQ_TOT_	0.45	0.37	0.44	0.41	0.36	0.33
IPAQ_LPA_	0.44	0.28	0.41	0.48	0.41	0.21
IPAQ_MPA_	0.33	0.15	0.35	0.37	0.31	0.03
IPAQ_VPA_	0.52	0.40	0.45	0.29	0.51	0.44

**PAQA**						

PAQA_TOT_	0.48	0.40	0.30	0.27	0.55	0.43
PAQA_LPA_	0.22	0.28	0.04	0.22	0.27	0.35
PAQA_MPA_	0.32	0.12	-0.18	0.00	0.56	0.23
PAQA_VPA_	0.57	0.40	0.59	0.23	0.51	0.43

### Criterion validity

Table 4 shows how the total amount of physical activity measured by the IPAQ and the PAQA positively correlates with the accelerometers. The correlation was considerably higher in urban areas compared to the rural. Analysis of dimensions of physical activity showed lower correlations for estimations of time spent on LPA and MPA for both the IPAQ and the PAQA.

**Table 4:** Criterion validity of the IPAQ and the PAQA (Spearman correlation coefficients)

	**All**	**Rural**	**Urban**
**IPAQ**			

IPAQ_TOT_	0.21	0.10	0.37
IPAQ_LPA_	0.14	0.09	0.21
IPAQ_MPA_	-0.01	0.01	-0.01
IPAQ_VPA_	0.29	0.33	0.28

**PAQA**			

PAQA_TOT_	0.27	0.13	0.41
PAQA_LPA_	0.20	0.29	0.10
PAQA_MPA_	0.18	-0.04	0.38
PAQA_VPA_	0.24	0.28	0.21

IPAQ_TOT _and PAQA_TOT _are expressed as MET-minutes.day^-1^, all other variables are time expressed in minutes

### Classification properties

Classification properties of repeated questionnaire registrations showed significant results for both the IPAQ and the PAQA (Table 5). Agreement between questionnaire and accelerometer classification was only significant for the IPAQ. Classification properties in urban areas were generally higher compared to rural. Classification agreement between the IPAQ and the PAQA was 52.4% (K = 0.29; P < 0.001).

**Table 5:** Classification agreement for test-retest and criterion validity (% agreement, Kappa statistic and significance level)

	**All**			**Rural**			**Urban**		
			
	**%** **agreement**	**K**	**P**	**% ** **agreement**	**K**	**P**	**%** **agreement**	**K**	**P**
			
Test-retest									
IPAQ	45.8%	0.19	<0.001	41.1%	0.09	0.13	50.0%	0.23	0.001
PAQA	46.7%	0.20	<0.001	38.6%	0.03	0.38	51.3%	0.25	0.001

Criterion validity									

IPAQ	39.7%	0.10	0.04	32.9%	0.00	0.51	47.9%	0.22	<0.001
PAQA	36.2%	0.04	0.24	32.8%	0.01	0.46	39.4%	0.10	0.12

### Feasibility

In general, the students found the questions quite complicated and needed close follow-up to fill the forms well. They found it particularly difficult to estimate average duration of activities on a weekly basis. The participants had no outstanding preference for the IPAQ or the PAQA. The average time to complete the IPAQ was approximately 10–15 minutes while this was 20–25 minutes for the PAQA. Students did not experience noteworthy problems wearing the accelerometers.

### Effect of stature and BMI

Standardizing our accelerometer outcomes for stature did not change the results of reliability and criterion validity. We also explored correlations using physical activity estimates standardised for BMI. The only effects that could be observed were distinct improvements in criterion validity for the estimations of time spent on LPA, ρ = 0.86 for IPAQ_LPA _and ρ = 0.49 for PAQA_LPA _and reliability coefficients of ICC = 0.54 for PAQA_TOT_.

## Discussion

Despite growing concern on nutritional status of adolescents worldwide and in developing countries, little is known on their physical activity patterns and energy expenditure. To our knowledge this is the first study examining the validity of physical activity questionnaires for adolescents in both rural and urban settings of a developing country and using an objective measure to assess criterion validity.

In general, the results of the present study show poor reliability of both the IPAQ and the PAQA and moderate correlation with the accelerometers. Our validity coefficients are generally lower than previously reported by other validation studies with adolescents [32-37].

Test-retest correlation of both questionnaires was poor and lower than most previous studies in developing countries. The IPAQ validation study, carried out with adults, reported test-rest correlations of ρ = 0.32 in rural South Africa and ρ = 0.25 in rural Guatemala, which was considerably lower compared to the overall reliability of ρ = 0.76 [17]. Sobngwi *et al *reported good reliability with Spearman correlation coefficients of ρ = 0.88 to ρ = 0.86 in urban and ρ = 0.96 to ρ = 0.93 in rural areas of Cameroun [38]. The Indian Physical Activity Questionnaire used in an urban population showed a reliability of ρ = 0.86 [39].

This study demonstrated moderate criterion validity with somewhat higher validity for the PAQA. Criterion validity in rural areas was poor for both questionnaires. We found only physical activity validation studies with adults in developing countries and none with adolescents. Criterion validity in rural South Africa was ρ = 0.46 [17]. A questionnaire for adults in rural Cameroun reported correlations with accelerometers of ρ = 0.60 to ρ = 0.74 for male and female adults [38]. Both studies however, used an interviewer-administered questionnaire in rural areas, which is likely to have improved their validity. A self-reported physical activity questionnaire in Pakistan with urban adult women showed good correlation (ρ = 0.60) with accelerometers [40].

Both the reliability and criterion validity for estimations of time spent on VPA was better compared to MPA and LPA. The considerable higher criterion validity for very intensive activity levels, similar to a previous study in adults, was observed [18]. Vigorous activities are easier to recall but are also prone to over reporting, as observed in the present study [41,42]. The overestimation of time spent on moderate and heavy activities however, would imply that the instruments cannot be used to classify individuals using absolute recommendations such as 30 minutes or 60 minutes per day. The criterion measure used in this study may have underestimated physical activity for particular activities, which may have induced the lower estimations of duration of MPA and VPA compared to the questionnaires [43,44]. This observation has been reported previously [37]. Accelerometers only record locomotor activities and they may consequently not capture all physical activities for children and adolescents well, in particular during free play [24,45]. Together with shuttle cock, badminton, running and martial arts, these activities were most frequently reported by the adolescents as vigorous and moderate activities in both rural and urban areas.

Classification properties, in particular for the IPAQ, reached statistical significance, but a lower Kappa value compared to other studies was obtained [37,46]. For specific use in adolescents living in rural areas however, these findings show that both questionnaires and application of the accelerometer requires further research.

The reasons for the low validity and differences between rural and urban areas remain unclear and cannot be answered by this study. There were no differences in supervision, personnel and material used in the rural and urban areas and in the first and second survey round. The questionnaires tested are not designed to give a detailed insight in the type of activities reported and important determinants that could affect the study results such as literacy rate, perception of the concepts studied, motivation, accuracy of recall, etc. were not assessed in the study.

## Conclusion

Our study showed that a questionnaire based on dimensions of physical activity and an activity-based questionnaire had a similar and limited validity for use in adolescents. Reliability was overall weak but criterion validity was acceptable. Both questionnaires however, showed overall poor validity and poor classification properties in rural areas. Our findings call for additional efforts to develop specific instruments to assess physical activity in adolescents in rural areas.

## Competing interests

The authors declare that they have no competing interests.

## Authors' contributions

LC, KP, KNC and KLNB designed the study. RV, ANDV and DNQ carried out the study and processed data in Vietnam. CL drafted the manuscript. HM processed and analysed the accelerometer data and KNC, ANDV, DNQ contributed to the analysis, interpretation and discussion of the study results. All authors critically revised the drafted manuscript.
